# Comparative Analysis of Cd Uptake and Tolerance in Two Mangrove Species (*Avicennia marina* and *Rhizophora stylosa*) with Distinct Apoplast Barriers

**DOI:** 10.3390/plants12223786

**Published:** 2023-11-07

**Authors:** Li-Fang Chang, Jiao Fei, You-Shao Wang, Xiao-Yu Ma, Yan Zhao, Hao Cheng

**Affiliations:** 1 South China Sea Institute of Oceanology, Chinese Academy of Sciences, Guangzhou 510301, China; 18843160398@163.com (L.-F.C.); feijiao@scsio.ac.cn (J.F.); yswang@scsio.ac.cn (Y.-S.W.); xiaoyuma613@gmail.com (X.-Y.M.); 2 College of Life Science and Agroforestry, Qiqihaer University, Qiqihaer 161006, China

**Keywords:** apoplast barriers, suberin, cadmium tolerance, mangrove

## Abstract

Mangrove plants demonstrate an impressive ability to tolerate environmental pollutants, but excessive levels of cadmium (Cd) can impede their growth. Few studies have focused on the effects of apoplast barriers on heavy metal tolerance in mangrove plants. To investigate the uptake and tolerance of Cd in mangrove plants, two distinct mangrove species, *Avicennia marina* and *Rhizophora stylosa*, are characterized by unique apoplast barriers. The results showed that both mangrove plants exhibited the highest concentration of Cd^2+^ in roots, followed by stems and leaves. The Cd^2+^ concentrations in all organs of *R. stylosa* consistently exhibited lower levels than those of *A. marina*. In addition, *R. stylosa* displayed a reduced concentration of apparent PTS and a smaller percentage of bypass flow when compared to *A. marina*. The root anatomical characteristics indicated that Cd treatment significantly enhanced endodermal suberization in both *A. marina* and *R. stylosa* roots, and *R. stylosa* exhibited a higher degree of suberization. The transcriptomic analysis of *R. stylosa* and *A. marina* roots under Cd stress revealed 23 candidate genes involved in suberin biosynthesis and 8 candidate genes associated with suberin regulation. This study has confirmed that suberized apoplastic barriers play a crucial role in preventing Cd from entering mangrove roots.

## 1. Introduction

Mangrove forests are a type of woody wetland community that thrive in the intertidal zone of tropical and subtropical coasts, enduring periodic inundation and possessing high productivity, return rate, decomposition rate, and resistance to adverse environmental conditions [1,2]. Due to the ability of mangroves to tolerate high levels of environmental pollutants, artificial mangrove wetlands have been proposed as a potential solution for treating urban wastewater [3]. Mangrove plants exhibit a certain degree of tolerance toward heavy metal; however, exceeding the maximum threshold will result in irreversible damage to the plant due to Cd stress [4,5]. The high activity and bioavailability of Cd can disrupt normal plant metabolism upon root absorption, leading to impaired photosynthesis, nutrient imbalance, and ultimately stunted growth [6,7]. Therefore, the presence of excessive heavy metals, such as cadmium (Cd), in sediment may impede the normal growth of mangrove plants [5]. *Avicennia marina* is widely distributed along the southeast coast of China and serves as a pioneer species in mangrove wetlands. Due to its high tolerance for heavy metals, *A. marina* can stabilize plants and has the potential to remediate heavy metal pollution in coastal wetlands [8,9]. *Rhizophora stylosa* also demonstrated high tolerance to metal pollution, showing low accumulation and even surpassing *A. marina* in terms of metal tolerances [10,11]. Previous studies have shown a significant positive correlation between metal tolerance and lignin/suberin contents of exodermis in mangrove plants [11,12]. Understanding the mechanism of heavy metal tolerance in mangroves is crucial for future conservation and restoration efforts.

The role of root apoplastic barriers and the radial apoplastic transport pathway play a key role in Cd translocation and accumulation in plants [13,14]. When water and ions move through the apoplastic pathway (also known as bypass flow), they are blocked by an apoplastic barrier in the endodermis or/and exodermis of plant roots [15]. The apoplastic barrier is a hydrophobic structure formed by lignin and suberin deposition, mainly composed of suberin lamellae with suberin as the main component [16]. Suberin is a hydrophobic secondary metabolite composed of phenolic compounds, glycerol, fatty acid derivatives, and primary fatty alcohols. It is typically deposited in specific tissues, such as root exodermis and endodermis, periderm, and other marginal tissues, to form suberization [17,18]. In addition to its deposition during normal development, the biosynthesis of suberin can also be induced by exposure to salt and Cd [13,19].

The mechanisms of suberin synthesis and Cd resistance in mangrove plants remain unclear. Studies have shown that mangrove plants with a higher tolerance to heavy metals possess a thicker suberin layer, which directly delays metal entry into the root and, consequently, contributes to a higher tolerance to heavy metals [11]. Currently, most studies on the correlation between apoplast barriers in mangrove plants and heavy metal absorption and transportation remain at a qualitative description stage [11,20,21]. The apoplastic flow from roots to shoots was traced using a fluorescent dye (trisodium-8-hydroxy-1,3,6-pyrenetrisulfonic acid; PTS), which is exclusively transported to xylem via the apoplast pathway under transpiration tension and can be employed for quantitative assessment of the strength of the apoplastic barrier [13,22]. This study specifically focused on tracing the apoplastic bypass flow to evaluate the effect of metal stress on Cd uptake.

This present study aims to achieve the following: (1) determine the relationship between Cd accumulation and the apoplast barrier, (2) elucidate the role of the apoplast barrier in Cd uptake and accumulation in mangrove plants, and (3) screen differentially expressed genes (DEGs) related to suberin synthesis, thereby exploring the molecular mechanisms underlying suberin synthesis and regulation in mangrove roots.

## 2. Results

### 2.1. Cd Uptake and Distribution in the Two Mangrove Cultivars

**Figure 1:**
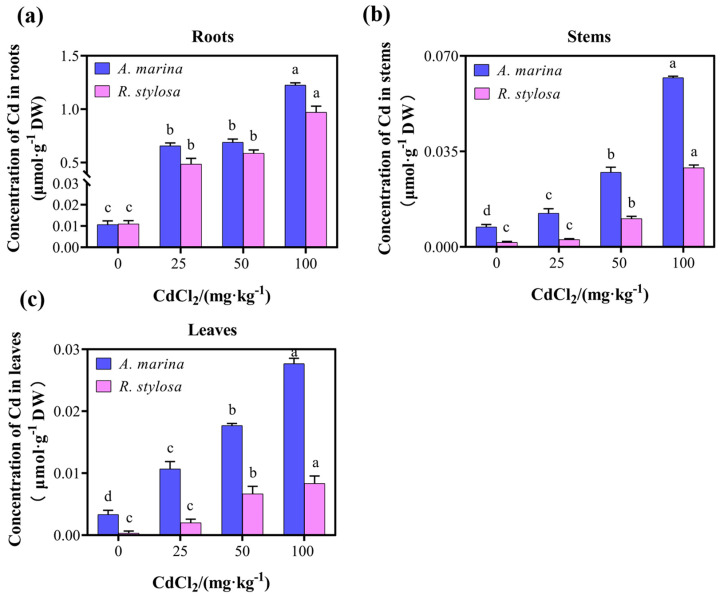
Cadmium uptake and distribution in seedlings of *A. marina* and *R. stylosa*. (**a**) Cd^2+^ ion concentration in the roots of control and treated seedlings. (**b**) Cd^2+^ ion concentration in the stems of control and treated seedlings. (**c**) Cd^2+^ ion concentration in the leaves of control and treated seedlings. The data presented are means ± SE from three biological replicates. Different letters within the same organ indicate significant differences between treatments as determined by one-way ANOVA (*p* < 0.05).

The biomass and chlorophyll content of both *A. marina* and *R. stylosa* exhibited significant suppression under cadmium stress, with a more pronounced inhibition observed as the concentration of cadmium increased (Figure S1). As shown in Figure 1, the distribution of Cd varied among the leaves, stems, and roots of *A. marina* and *R. stylosa* seedlings. The concentration of Cd^2+^ in each organ of both mangrove plants was relatively low when CdCl_2_ was not applied. However, with increasing concentrations of CdCl_2_ treatment, the Cd^2+^ concentrations also significantly increased in plant organs. The distribution pattern of Cd in the organs of the two mangrove plants followed this order: root (Figure 1a) > stem (Figure 1b) > leaf (Figure 1c). It is worth noting that the concentration of Cd^2+^ in each organ of *R. stylosa* was lower than that in *A. marina* under identical CdCl_2_ treatment.

### 2.2. Apoplastic Bypass Flow and Apparent PTS Content in the Two Mangrove Cultivars

**Figure 2:**
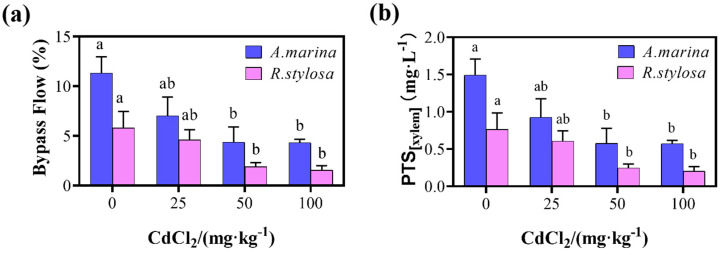
Apoplastic bypass flow (**a**) and apparent PTS (trisodium-8-hydroxy-1,3,6-pyrenetrisulfonic acid) content (**b**) in the *A. marina* and *R. stylosa* under cadmium stress. (**a**) Apoplastic bypass flow in the *A. marina* and *R. stylosa* under cadmium stress. (**b**) The apparent PTS content in the *A. marina* and *R. stylosa* under cadmium stress. Different letters within the same organ indicate significant differences between treatments as determined by one-way ANOVA (*p* < 0.05). Different letters within the same organ indicate significant differences between treatments as determined by one-way ANOVA (*p* < 0.05).

Compared with the CK, the percentage of bypass flow and PTS concentration of Cd-treated *A. marina* and *R. stylosa* were significantly reduced, as depicted in Figure 2. Furthermore, with the gradual increase in Cd^2+^ concentration, the percentage of bypass flow and PTS concentration of both mangrove species also decreased gradually. In *A. marina*, the percentage of bypass flow decreased by 38.07%, 61.31%, and 61.75%, respectively, under different Cd^2+^ concentration treatments compared to the control group, while the apparent PTS concentrations also exhibited a decrease of 37.58%, 61.07%, and 61.74%, respectively. In *R. stylosa*, the percentage of bypass flow decreased by 20.52%, 67.41%, and 73.28%, respectively, compared to the control group, while the apparent PTS concentrations reduced by 19.74%, 67.11%, and 73.68%, respectively. Additionally, in both control and Cd-treated groups, *R. stylosa* exhibited lower percentages of bypass flow and PTS concentration compared to *A. marina*. 

### 2.3. Root Anatomical Characteristics of Suberin Lamellae in Response to Cd Treatment

**Figure 3:**
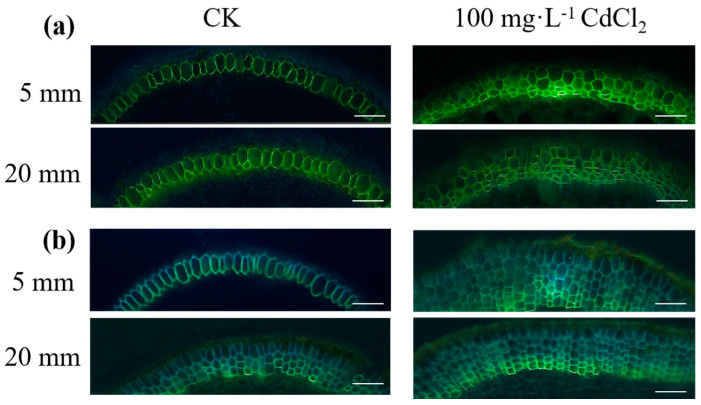
Deposition of suberin lamellae (SL) in the endodermis of *A. marina* (**a**) and *R. stylosa* (**b**) was observed under 0 and 100 mg·L^−1^ CdCl_2_, at distances of 5 mm and 20 mm from the root tip. Sections were stained with a solution containing 0.01% (*w*/*v*) Fluorol Yellow 088 for 1 h, followed by observation using a fluorescence microscope. The scale bar represents a length of 50 μm.

In order to investigate the root anatomical features and exodermal lignification/suberization between the two mangrove plants, the staining pattern of suberin lamellae in root cells was generated by Fluorol Yellow 088 at the root tips in the endodermis. As illustrated in Figure 3, both *A. marina* and *R. stylosa* exhibited weaker suberization closer to the root tip. At a distance of 5 mm from the tip, suberization was lower than that at 20 mm from the tip. At the same position, *R. stylosa* showed a higher degree of suberization compared to *A. marina*. Additionally, Cd treatment significantly enhanced endodermal suberization in both *A. marina* and *R. stylosa* roots. 

### 2.4. Net Fluxes of Cd^2+^ in Roots Surface 

**Figure 4:**
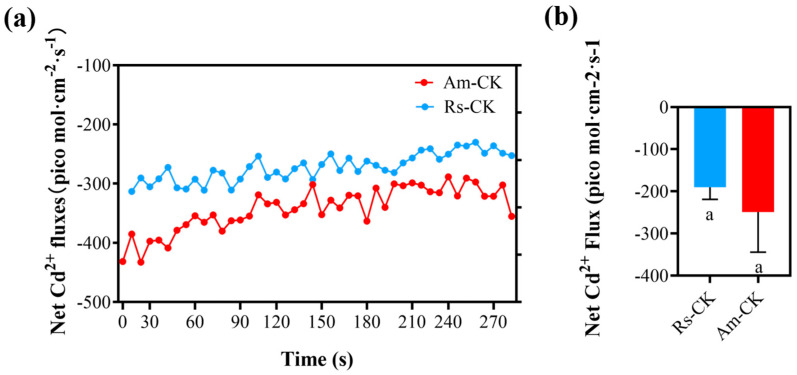
Net Cd^2+^ fluxes in coniferous zone of *A. marina* and *R. stylosa* roots under cadmium stress. The negative values represent Cd^2+^ influx into the root from the test solution. (**a**) The temporal variation of Net Cd^2+^ fluxes during the experimental period was quantified using the noninvasive microtest technique (NMT). (**b**) The average value of Net Cd^2+^ fluxes was calculated on temporal variation value. The letter "a" denotes that there is no statistically significant difference between the two datasets as determined by one-way ANOVA (*p* < 0.05).

The negative values represent the influx of Cd^2+^ into the root from the test solution. As depicted in Figure 4, high Cd^2+^ fluxes were detected in the coniferous zone of both *A. marina* and *R. stylosa* under control conditions, and *A. marina* exhibited higher net Cd^2+^ fluxes than *R. stylosa*.

### 2.5. Identification and Functional Classification of DEGs

**Figure 5:**
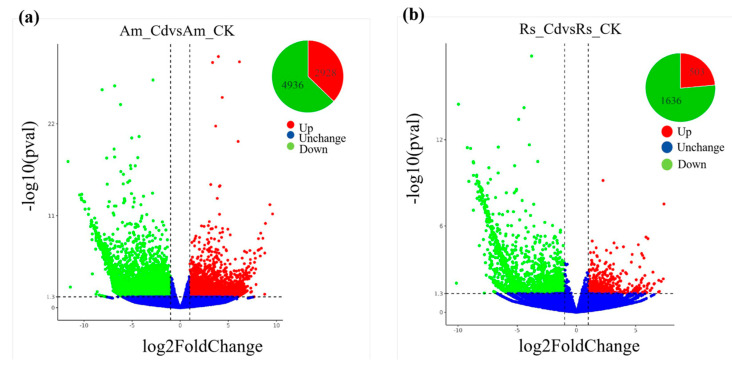
Number of DEGs (differentially expressed genes) in *A. marina* (**a**) and *R. stylosa* (**b**) under cadmium stress.

**Figure 6:**
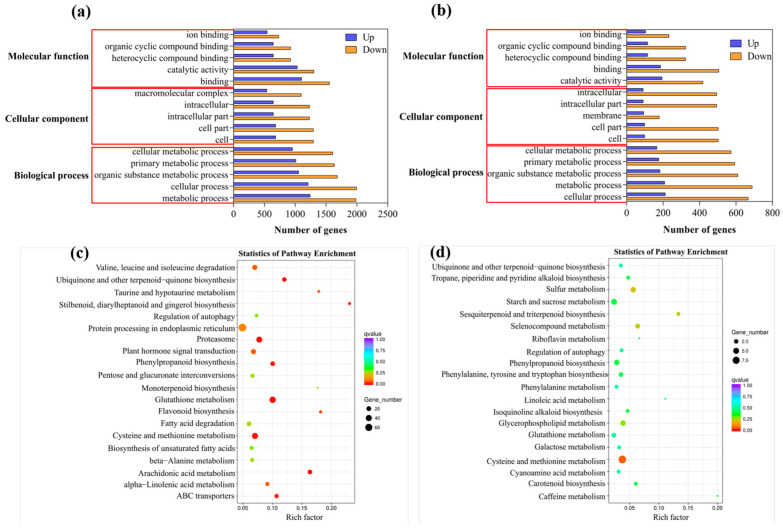
Gene Ontology (GO) enrichment analysis was performed on differentially expressed genes (DEGs) of *A. marina* (**a**) and *R. stylosa* (**b**), while Kyoto Encyclopedia of Genes and Genomes (KEGG) enrichment analysis was conducted on DEGs of *A. marina* (**c**) and *R. stylosa* (**d**).

As illustrated in Figure 5, the results demonstrated that *A. marina* roots exhibited 2928 up-regulated genes and 4936 down-regulated genes, while *R. stylosa* roots displayed 503 up-regulated genes and 1636 down-regulated genes in response to Cd treatment. As shown in Figure 6a, the GO enrichment analysis of DEGs in *A. marina* revealed that up-regulated genes are significantly enriched in GO terms related to binding, the metabolic process, and the cell and cellular process after Cd stress. In contrast, the down-regulated DEGs are significantly enriched in GO terms associated with metabolism, cellular processes, binding, and catalytic activity. The GO enrichment analysis of DEGs in *R. stylosa*, as shown in Figure 6b, reveals a significant enrichment of up-regulated genes in GO terms related to catalytic activity, binding, and cellular processes. Conversely, down-regulated genes are notably enriched in GO terms associated with metabolic processes, cellular processes, binding, and catalytic activity. Figure 6c displays the results of KEGG enrichment analysis for DEGs in *A. marina*, revealing that a total of 1250 genes are involved in 108 metabolic pathways. Among these, the most highly expressed genes were found to be associated with protein processing in endoplasmic reticulum and glutathione metabolism, with 73 and 43 genes, respectively. Additionally, phenylpropanoid biosynthesis, ABC transporters, phenylalanine metabolism, cutin, suberin, and wax biosynthesis, as well as fatty acid elongation, were also enriched with the number of enriched genes being 18, 15, 9, 3, and 2, respectively. The results of KEGG enrichment analysis for DEGs in *R. stylosa* are shown in Figure 6d. A total of 1380 genes were found to be involved in 65 metabolic pathways. The most highly expressed genes were related to ribosome, cysteine, and histidine metabolism, with 12 and 9 genes, respectively. Furthermore, phenylalanine biosynthesis, peroxisome, phenylalanine metabolism, and ABC transporter were also enriched with four, three, two, and one gene(s), respectively.

### 2.6. Candidate Genes for Suberin Biosynthesis and Regulation

**Figure 7:**
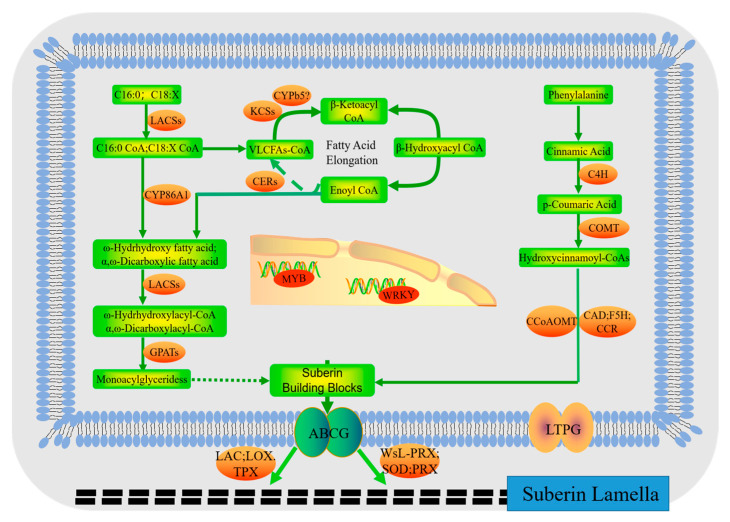
Hypothetical metabolic pathway for suberin biosynthesis in mangrove plants.

The up-regulated DEGs in the transcriptome were annotated in NR and other databases to obtain gene function annotations. Based on their functions, they were classified into three categories: synthesis of suberin monomers, polymerization and assembly of suberin monomers, and transcription factors involved in suberin synthesis and regulation (Table 1). According to this speculated gene function, a molecular synthesis mechanism map of suberin was depicted in Figure 7.

## 3. Discussion

### 3.1. Role of Suberin on Cd^2+^ Uptake, Transportation, and Tolerance in Mangrove Seedlings

Cd is a nonessential trace metal that exhibits high toxicity in almost all living organisms [23]. Due to its elevated activity and bioavailability, Cd can impede plant growth [24]. In this present study, we observed the induced formation of hydrophobic barriers near the root tips in the endodermis and exodermis following Cd treatment in *R. stylosa* and *A. marina* seedlings, which aligns with previous findings in rice [13]. After Cd stress treatment, both *R. stylosa* and *A. marina* showed a decrease in bypass flow and Cd^2+^ flux, indicating that appropriate Cd treatment can enhance plants’ apoplastic barrier and tolerance to this metal, which is consistent with the findings for *Populus cathayana* [25]. This present study presents novel findings that the exosomal barrier in mangroves effectively hinders the absorption and translocation of heavy metal Cd, thereby resulting in reduced concentrations of CdCl_2_ in treated plants. In addition, the exoplasmic barrier is related to the root suberization of plants. These findings provide valuable insights and guidance for screening and breeding of high-tolerance plants.

Suberin acts as a physical barrier when deposited in the endodermis or exodermis of plant roots, preventing water and nutrient losses from the tissues it surrounds, as well as providing protection against environmental stresses, such as pathogens, drought, and salt stress [26,27,28,29]. As a crucial protective barrier for roots, phellem not only regulates ion absorption and transportation but also plays a significant role in the response mechanism to heavy metal stress in mangrove plants [11]. The apoplastic tracer PTS is nontoxic to plants and can only be transported exclusively through the apoplastic pathway to the shoot. The lower the apparent PTS concentration and percentage of bypass flow, the lower the concentration of heavy metals flowing into the plant, resulting in a stronger ability of the exocytosomal barrier [13,30]. In this study, *R. stylosa* showed a higher degree of suberization compared to *A. marina* at the same position, leading to a stronger exoplasmic barrier to the absorption and transport of Cd^2+^ and stronger resistance to heavy metals. Previous research has shown that root exodermis with a high degree of suberization exhibits greater tolerance to heavy metals [11,31]. The stronger the apoplastic barrier, the less PTS is transported to aboveground and the lower the bypass flow rate. In this study, *R. stylosa* exhibited a lower initial bypass flow than *A. marina*, indicating a stronger initial apoplastic barrier and Cd tolerance in *R. stylosa*.

### 3.2. Effects of Cd^2+^ Stress on Suberin Biosynthesis in Mangrove Plants

This study revealed that *R. stylosa* exhibited greater resistance to heavy metals compared to *A. marina*, which is consistent with previous research [11]. The heavy metal tolerance of mangrove plants was found to be positively correlated with their lignin/suberin content [11,12]. In the transcriptome data of this study, genes associated with the biosynthesis and assembly of suberin were identified in both *A. marina* and *R. stylosa* (Table 1), indicating the involvement of suberin in the response to heavy metal stress in mangrove plants.

Suberin, a hydrophobic secondary metabolite composed of phenols, glycerol, fatty acid derivatives, and primary fatty alcohols, is typically deposited on the cell walls of specific tissues, including the root endodermis, exodermis, peridermis, and other marginal tissues, resulting in the formation of phellem [19,32]. The deposition of suberin primarily occurs on the secondary cell wall, while its polymerization mechanism remains unclear [33]. Scientists have obtained a large number of genes through transcriptome technology using *Arabidopsis thaliana* and other model plants [32]. The cadmium-transporter genes of mangrove plants were illustrated to improve the Cd tolerance of transgenic plants [9]. In this study, we conducted a comprehensive transcriptomic analysis on the roots of *R. stylosa* and *A. marina* under Cd stress to explore the molecular synthesis mechanism of suberin in mangrove plants. 

There are two prerequisite substances for suberin monomers: very long-chain fatty acid (VLCFA) precursors and phenylalanine. When VLCFAs are used as precursors, the elongation of plastid-derived fatty acids (FAs) is the initial step in the biosynthesis of such precursors [34], which is catalyzed by LACS and accomplished through the FAE complex [35]. The FAE complex is composed of four enzymes: KCS, β-ketoacyl-CoA reductase (KCR), β-hydroxyacyl-CoA dehydratase (HCD), and enoyl-CoA reductase (ECR) [36]. Under Cd stress, the expression of *KCS1* and *LACS6* are up-regulated in *A. marina,* indicating that the elongation of FAs was promoted. CER1 and CER3 encode core components of a redox-dependent multienzyme complex, which can interact with electron-transferring cytochrome b5 hemoproteins (CYTB5s), to act as cofactors to facilitate the very long-chain fatty acid to produce VLC-alkanes [32,37,38]. CYP86A1 encodes a fatty acid ω-hydroxylase that catalyzes the ω-site hydroxylation to produce ω-hydroxy acids [39]. GPATs catalyze acyl-CoA or acyl-ACP to produce lysophosphatidic acids (LPAs), which are reduced to suberin monomers. GPAT5 is specifically involved in suberin biosynthesis in seed coats and root tissues [40,41]. In this study, *CER1*, *CER3*, *CYTB5*, *CYP86A1*, and *GPAT5* are up-regulated in *A. marina*, suggesting that suberin biosynthesis with VLCFA as a precursor was increased. 

When phenylalanine serves as the precursor, it is oxidized to form cinnamate and then parahydroxylated by C4H/CYP73A to yield p-coumaric acid [42]. CCoAOMT functions as a typical O-methyltransferase protein, producing Ferulate-CoA. F5H/CYP84A and COMT can generate the 4-hydroxy-3,5-dimethoxy-substituted hydroxycinnamate structure of sinapic acid in various species. CCR and CAD are capable of converting hydroxycinnamoyl-CoA thioesters into corresponding monoxylitol [32,43]. The expression of *C4H*/*CYP73A,* F5H/CYP84A, and *CCoAOMT* were up-regulated in *A. marina*; as well as this, *COMT* and *CAD* are up-regulated in both *A. marina and R. stylosa*, *indicating* that suberin biosynthesis with phenylalanine as a precursor was increased. 

To form suberin, the monomers synthesized in the membrane must be transported out of the plasma membrane (PM) for polymerization and assembly. Several transporters including AtABCG1, AtABCG2, AtABCG6, AtABCG11, AtABCG20, and OsABCG5 are involved in this process [44,45,46,47]. Lipid transfer proteins (LTPs) facilitate the transportation of cuticle precursors from the plasma membrane to the cell wall surface [48]. LTPG2 has been confirmed to participate in cutin transport, and the transportation of cutin and cork shares common elements [49]. Here, the expressions of *ABCG11* and *LIPG2* are found to be up-regulated in *A. marina*, indicating that transportation of suberin monomers is also promoted.

The polymerization and assembly of suberin are primarily catalyzed by oxidases. In tomatoes, TPX1 is exclusively expressed in cells undergoing lignin and suberin synthesis [50,51]. Meanwhile, PRX4, SOD, and LAC1 had been speculated to be implicated in suberin biosynthesis [52,53,54]. WsL-PRX, belonging to the same oxidase class, may have similar functions in lignin formation [55]. *TPX1*, *PRX4*, *LAC7*, *LAC14*, *SOD1*, and *SOD2* are up-regulated in *A. marina* or both in *R. stylosa*, indicating these genes are involved in the polymerization and assembly of suberin. LOX1 is involved in synthesizing jasmonic acid, which induces suberization and is speculated to be associated with suberin production [56]. *LOX1* is up-regulated in both *A. marina and R. stylosa,* implying that the synthesis of suberin was promoted.

Transcription factors (TFs) regulate gene expression and participate in plant stress response by binding to specific target gene sequences. MYB, WRKY, MYC, and NAC are the main TFs involved in suberin synthesis regulation. MYB53 acts downstream of the ABA signaling pathway and induces suberin biosynthesis in the endodermis [57]. MYB39 is a positive transcription factor that promotes suberin deposition in the root endodermis layer. The transient expression of MYB39 in *N. benthamiana* leaves leads to the accumulation of major suberin monomers and the deposition of suberin-like lamellae [29,32,58]. WRKY33 serves as the upstream regulatory transcription factor of CYP94B1, which is involved in suberin biosynthesis [59]. In this study, *MYB53* and *MYB39* are up-regulated in *A. marina*, and *WRKY33* is up-regulated in *R. stylosa*, indicating that these genes are involved in suberin synthesis.

Overall, these results indicated that Cd stress induced the expression of genes involved in the synthesis, transport, and assembly of suberin, which deepened our understanding of heavy metal tolerance mechanisms in mangrove plants and provided a reference for genetic engineering of plant heavy metal resistance.

## 4. Materials and Methods

### 4.1. Plant Materials and Treatments

The propagules of *A. marina* and *R. stylosa* were collected from Beihai City, Guangxi Province, China. Healthy propagules of uniform size were carefully selected and watered with the 1/2 Hoagland solution. After *A. marina* and *R. stylosa* grew two pairs of leaves, the seedlings were transferred into new pots with soil. The soil for growing plants was obtained from the identical geographical region as the mangrove seedlings. The soil was sifted through an 8 mm diameter screen to ensure uniform particle size, and then exposed to cadmium pollution at concentrations of 0, 25, 50, and 100 mg·kg^−1^, respectively, corresponding to the CK treatment, low concentration treatment, medium concentration treatment, and high concentration treatment. After thorough mixing, the soil was used to transplant the seedlings. Then, the mangrove seedlings were carefully transferred to an artificial climate incubator for cultivation and irrigated with a 1/2 Hoagland nutrient solution every three days. The culture conditions were as follows: 25 °C, 14/10 h light/dark cycle, 75% relative humidity, and 20,000 LX illumination intensity. After 4 weeks, seedlings treated with Cd were harvested and washed with deionized water to eliminate surface Cd, and then separated into leaves, stems, and roots to analyze Cd^2+^ concentration. 

### 4.2. Measurement of Total Ion Concentration (Cd^2+^) from Plants

The samples were dried in an oven at 70 °C for 4 days and digested with concentrated nitric acid (HNO_3_). The level of Cd^2+^ in the acid-digested samples was determined using inductively coupled plasma-mass spectrometry (ICP-MS; Perkin Elmer NexION 2000, Waltham, MA, USA).

### 4.3. Measurement of Apoplastic Bypass Flow of Different Cultivars

Three seedlings of each of *A. marina* and *R. stylosa* treated above were subjected to treatment with 100 mg·L^−1^ PTS, a tracer for apoplastic bypass flow, using the method described [13]. The plants were allowed to undergo a 96-h period of PTS absorption, and then transferred to a nutrient solution without PTS absorption for 48 h, thereby ensuring all absorbed PTS was transferred to the upper parts. The weight difference is used to calculate plant transpiration. Stems and leaves were harvested and dried in an oven at 70 °C until constant weight was achieved. Subsequently, samples were weighed and extracted with 8 mL of ultrapure water at 90 °C for 2 h. The PTS fluorescence in the extract was quantified using a microplate reader (Cytation 5, BioTek, Winooski, VT, USA) with excitation at 403 nm and emission at 510 nm.

The percentage of bypass flow can be calculated using the following formula:

$$PTS[xyl]=\frac{\mathrm{PTS}\left[\mathrm{stem}\right]+\mathrm{PTS}[\mathrm{leaves}]}{\mathrm{Water}\;\mathrm{transpiration}\;\mathrm{volume}},Bypass\;flow(\%)=\frac{\mathrm{PTS}[\mathrm{xyl}]}{\mathrm{PTS}[\mathrm{ext}]}\times 7.57\times 100,$$

where PTS[ext] represents the concentration of PTS in the external solution, and 7.57 is an empirical correction factor accounting for the relative mobility of PTS and water.

### 4.4. Histochemical Detection of Suberin Lamellae (SL) in Roots

The adventitious roots of treated plants were selected, and cross sections were meticulously prepared from the 10% region of total root length. To visualize suberin lamellae by fluorescent microscopy, a histological staining procedure with the dyes Fluorol Yellow 088 was applied to plant organs [60]. The suberin lamellae sections were then stained with Fluorescent Yellow 088 (0.1%, *w*/*v*) for 2 h in complete darkness before observation under a fluorescence microscope using UV light.

### 4.5. Measurement of Cd^2+^ Fluxes 

The mangrove seedlings were washed with pure water to remove any Cd and NaCl residue and then transferred carefully to pots for cultivation with a mixture of 1/2 Hoagland nutrient solution and 50 mg·L^−1^ CdCl_2_ every three days. The culture conditions were set at 25 ℃, with a 14/10 h light/dark cycle, 75% relative humidity, and 20,000 LX illumination intensity. After 4 weeks, samples were collected and used for the measurement of Cd^2+^ fluxes. Net fluxes of Cd^2+^ were measured using the noninvasive microtest technique (NMT) (NMT100 Series; Younger USA, Amherst, MA, USA) and _I_F_LUXES_/_IM_F_LUXES_ 1.0 software (Younger USA, Amherst, MA, USA), which is capable of integrating and coordinating differential voltage signal collection, motion control, and image capture simultaneously. The Cd-microelectrode needs to be calibrated before measuring Cd^2+^ flux. The primary roots of intact seedlings were rinsed with deionized water, immobilized, and equilibrated for 10 min in the measuring solution (0.25 mM CdCl_2_, pH 6.0). The root was then used to measure the point of peak flow rate at a distance of 500 µm. Each treatment had 8 biological replicates.

### 4.6. RNA-Seq Library RNA Preparation, Sequencing, and Analysis

Healthy propagules of uniform size were selected and cultivated in an artificial climate incubator until *A. marina* and *R. stylosa* grew two pairs of leaves. The mangrove seedlings were then carefully transferred to a new plot filled with 1/2 Hoagland’s solution and allowed to acclimate for two days. Subsequently, the plants were exposed to 50 mg·L^−1^ CdCl_2_ in 1/2 Hoagland’s solution, defined as Cd Group. Seedlings treated only with 1/2 Hoagland’s solution were set as CK group. After 3 days, the roots of seedlings were harvested. Total RNA was extracted using the Tiangen RNAprep Pure polysaccharide polyphenol plant total RNA extraction kit (TIANGEN, Beijing, China). After assessing the concentration and integrity of total RNA, Poly (A)-tailed mRNA was enriched from the total RNA using oligomeric (dT) magnetic beads, followed by random division of bivalent cations in the buffer. The fragmented mRNA was utilized as a template for the synthesis of first-strand cDNA, employing random oligonucleotides as primers by M-MuLV reverse transcriptase system. Subsequently, RNase-H was employed to degrade the RNA strand, followed by synthesis of the second cDNA strand utilizing a DNA polymerase I system. The resulting double-stranded cDNA was purified, repaired at the ends, appended with a tail, and connected to a sequencing adapter. Suitable fragments (370–420 bp) were screened by agarose gel electrophoresis and enriched by PCR to construct cDNA libraries. The library quality was tested before sequencing on an Illumina HiSeq platform (Illumina, San Diego, CA, USA).

### 4.7. Statistical Analysis

All data were analyzed using SPSS 21.0 (IBM Company, Armonk, NY, USA). Each determination was performed in triplicate, and the results are presented as mean values ± standard error (SE). One-way analysis of variance (ANOVA), Least-Significant Difference (LSD), and Tamhane’s T2 tests were used to determine the significance of treatments and control groups (*p* < 0.05).

## 5. Conclusions

The findings indicate that suberized apoplastic barriers in roots play a crucial role in excluding Cd. Species with stronger barriers had less bypass flow, resulting in a more effective reduction in Cd transfer into upper parts via the apoplastic pathway. Under Cd stress, the corking-induced apoplastic barrier is enhanced. The extent of both initial formation and response to Cd stress of apoplastic barriers determines the effectiveness of these barriers. Therefore, the *R. stylosa* exhibits a stronger initial apoplastic barrier compared to *A. marina*. Due to the hydrophobic barrier of suberin that excludes Cd at roots, enhancing its formation can improve plant Cd tolerance. After analyzing the root transcriptome of *R. stylosa* and *A. marina* under Cd stress, we have successfully identified 23 candidate genes associated with suberin synthesis using very long-chain fatty acid (VLCFA) and phenylalanine as precursors, as well as eight candidate genes involved in suberin regulation through nuclear transcription factors. Based on this speculated gene function, a molecular synthesis mechanism map of suberin was constructed. This study deepens our understanding of heavy metal tolerance mechanisms in mangrove plants, as well as facilitating screening for stress-resistant mangrove species, thereby providing a theoretical basis for coastal mangrove protection and restoration.

## Figures and Tables

**Table 1:** Key steps in the biosynthesis and assembly of suberin.

Gene	Corresponding Enzyme Function ^1^	Gene ID	Plant Species
Synthesis of suberin monomers			
*LACS6*	Long-chain acyl-CoA synthetase	Cluster-29888.0	*A. marina*
*KCS1*	3-ketoacyl-CoA synthase	Cluster-14650.20981	*A. marina*
*CER1*	Very-long-chain aldehyde decarbonylase	Cluster-14650.23822	*A. marina*
*CER3*	Very-long-chain aldehyde decarbonylase	Cluster-12850.10147	*R. stylosa*
*GPAT5*	Glycerol-3-phosphate acyltransferase	Cluster-14650.34157	*A. marina*
*CYP86A1*	Cytochrome P450-dependent fatty acid ω -hydroxylase	Cluster-14650.26258	*A. marina*
*CYTB5*	Cytochrome b_5_	Cluster-14650.28547	*A. marina*
*CCR1*	Cinnamoyl-CoA reductase	Cluster-14650.33944	*A. marina*
*CCR*	Cinnamoyl-CoA reductase	Cluster-12850.9370	*R. stylosa*
*F5H*/*CYP84A*	Ferulate 5-hydroxlyase	Cluster-14650.33388	*A. marina*
*COMT*	Caffeic acid O-methyltransferase	Cluster-14650.15351	*A. marina*
*COMT*	Caffeic acid O-methyltransferase	Cluster-3477.0	*R. stylosa*
*CAD*	Cinnamyl alcohol dehydrogenase	Cluster-55339.0	*A. marina*
*CAD*	Cinnamyl alcohol dehydrogenase	Cluster-55339.0	*R. stylosa*
*C4H*/*CYP73A*	Cinnamic acid 4-hydroxylase	Cluster-14650.32547	*A. marina*
*CCoAOMT*	Caffeoyl-CoA-O-methyltransferase	Cluster-14650.32082	*A. marina*
Polymerization and assembly of suberin monomer			
*LTPG2*	Nonspecific lipid transfer protein GPI-anchored 2	Cluster-14650.39736	*A. marina*
*ABCG11*	ATP-binding cassette subfamily G transporter	Cluster-14650.27512	*A. marina*
*PRX4*	Peroxygenase	Cluster-14650.16427	*A. marina*
*TPX1*	Cationic peroxidase	Cluster-14650.35143	*A. marina*
*TPX1*	Cationic peroxidase	Cluster-12850.633	*R. stylosa*
*WsL-PRX*	Lignin-forming anionic peroxidase	Cluster-14650.24652	*A. marina*
*WsL-PRX*	Lignin-forming anionic peroxidase	Cluster-12850.8199	*R. stylosa*
*LOX1*	Lipoxygenase	Cluster-12850.12299	*R. stylosa*
*LAC7*	Laccase	Cluster-14650.28143	*A. marina*
*LAC14*	Laccase	Cluster-14650.29125	*A. marina*
*SOD2*	Superoxide dismutase, Fe-Mn family	Cluster-52365.0	*A. marina*
*SOD1*	Superoxide dismutase, Cu-Zn family	Cluster-14650.35925	*A. marina*
Transcription factors (TF)			
*MYB53*	Transcription factor MYB	Cluster-14650.15097	*A. marina*
*MYB39*	Transcription factor MYB	Cluster-14650.18329	*A. marina*
*WRKY33*	Transcription factor WRKY	Cluster-12850.13359	*R. stylosa*

^1^ The results was annotated based on gene function.

## Data Availability

All analyzed or generated data are included in this article. The data analyzed or generated in this study can be obtained from the corresponding author upon reasonable request.

## Supplementary Materials

The following supporting information can be downloaded here: https://www.mdpi.com/article/10.3390/plants12223786/s1, Figure S1: The biomass (a) and chlorophyll content (b) of both *A. marina* and *R. stylosa* under cadmium stress.
