# Supplementary material for: Comparative Analysis of Cd Uptake and Tolerance in Two Mangrove Species (Avicennia marina and Rhizophora stylosa) with Distinct Apoplast Barriers
Source: Plants (Basel). 2023 Nov 7;12(22):3786. doi: 10.3390/plants12223786 (PMC10674663; doi:10.3390/plants12223786)
Supplement: Supplementary file 1 [file plants-12-03786-s001.zip › plants-2686290-supplementary.pdf]

Supplementary Materials

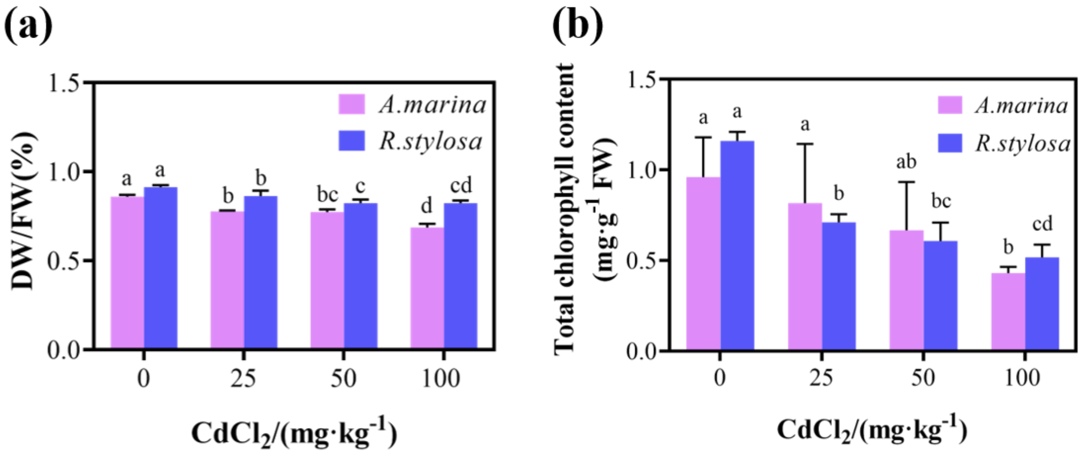

Figure S1. The biomass (**a**) and chlorophyll content (**b**) of both *A. marina* and *R. stylosa* under cadmium stress. DW = dry weight (g), Fw = fresh weight (g). Different letters within the same organ indicate significant differences between treatments as de-termined by one-way ANOVA ($p <$ 0.05).
